# Posterior mediastinal ganglioneuroma with peripheral replacement by white and brown adipocytes resulting in diagnostic fallacy from a false-positive 18F-2-fluoro-2-deoxyglucose-positron emission tomography finding: a case report

**DOI:** 10.1186/1752-1947-8-345

**Published:** 2014-10-15

**Authors:** Kenji Yorita, Akihiro Yonei, Takanori Ayabe, Hiroshi Nakada, Ken Nakashima, Tsuyoshi Fukushima, Hiroaki Kataoka

**Affiliations:** 1 Section of Oncopathology and Regenerative Biology, Department of Pathology, Faculty of Medicine, University of Miyazaki, 5200 Kihara, Kiyotake, Miyazaki 889-1692, Japan; 2 Department of Cardiovascular, Thoracic and General Surgery, Faculty of Medicine, University of Miyazaki, 5200 Kihara, Kiyotake, Miyazaki 889-1692, Japan; 3 Department of Radiology, Faculty of Medicine, University of Miyazaki, 5200 Kihara, Kiyotake, Miyazaki 889-1692, Japan; 4 Department of Surgery, Koga General Hospital, 1749-1 Sudaki, Ikeuchi-cho, Miyazaki 880-0041, Japan

**Keywords:** Brown adipocyte, FDG-PET imaging, Ganglioneuroma, Posterior mediastinum

## Abstract

**Introduction:**

Ganglioneuroma is a rare tumor in the posterior mediastinum; fat-containing ganglioneuromas are rarely reported. The present case report documents a brown fat-containing, posterior mediastinal ganglioneuroma, which has not been reported previously. Radiological examination, in particular 18F-2-fluoro-2-deoxyglucose-positron emission tomography, suggested that the tumor had low-grade malignant potential. This led to uncertainty at preoperative diagnosis.

**Case presentation:**

An asymptomatic 66-year-old Japanese woman with no significant past medical history was referred for the evaluation of a posterior mediastinal mass. Although its size had not changed in the past 5 years, a malignant lipomatous tumor could not be excluded due to the presence of intratumoral fat and increased 18F-2-fluoro-2-deoxyglucose uptake observed by positron emission tomography imaging. A computed tomography-guided core-needle biopsy revealed a mixture of mature adipocytes, spindle-shaped cells, and fibrotic stroma. Definite diagnosis was not possible, and surgical resection was performed. Three years after the surgery, she remains disease-free.

**Conclusions:**

Histological diagnosis of the surgically resected mass confirmed ganglioneuroma with substantial amounts of white and brown adipose tissues in peripheral areas. The existence of both ganglion cells and brown fat tissue intensified the accumulation of 18F-2-fluoro-2-deoxyglucose, resulting in a false-positive result by positron emission tomography. Considering this, ganglioneuroma should not be excluded either clinically or pathologically in fat-containing, posterior mediastinal tumors.

## Introduction

Ganglioneuroma is a rare, benign neurogenic tumor predominantly found in adolescents older than 10 years of age or young adults [1]. Most ganglioneuromas are thought to develop *de novo* rather than by maturation of a preexisting neuroblastoma, and are usually found in the posterior mediastinum and retroperitoneum. Some symptomatic cases with hypertension, watery diarrhea, or virilization have been reported [2], although ganglioneuromas are usually asymptomatic. On histological examination a ganglioneuroma is composed of mature ganglion cells, Schwann cells, and nerve fibers. A ganglioneuroma with abundant adipose tissue is unusual, and only five cases have reported in the English and Japanese literatures to date, and were referred to as ganglioneuroma with fatty replacement or lipomatous ganglioneuroma [3-7]. Here a case of ganglioneuroma with fatty replacement is reported. The interest in the present case lies in the finding that the tumor contained substantial amounts of brown adipose tissue intermingled in the neoplastic mass. The existence of ganglion cells and brown fat tissue probably resulted in a false-positive signal with 18F-2-fluoro-2-deoxyglucose (FDG)-positron emission tomography (PET), which caused uncertainty in preoperative diagnosis.

## Case presentation

**Figure 1:**
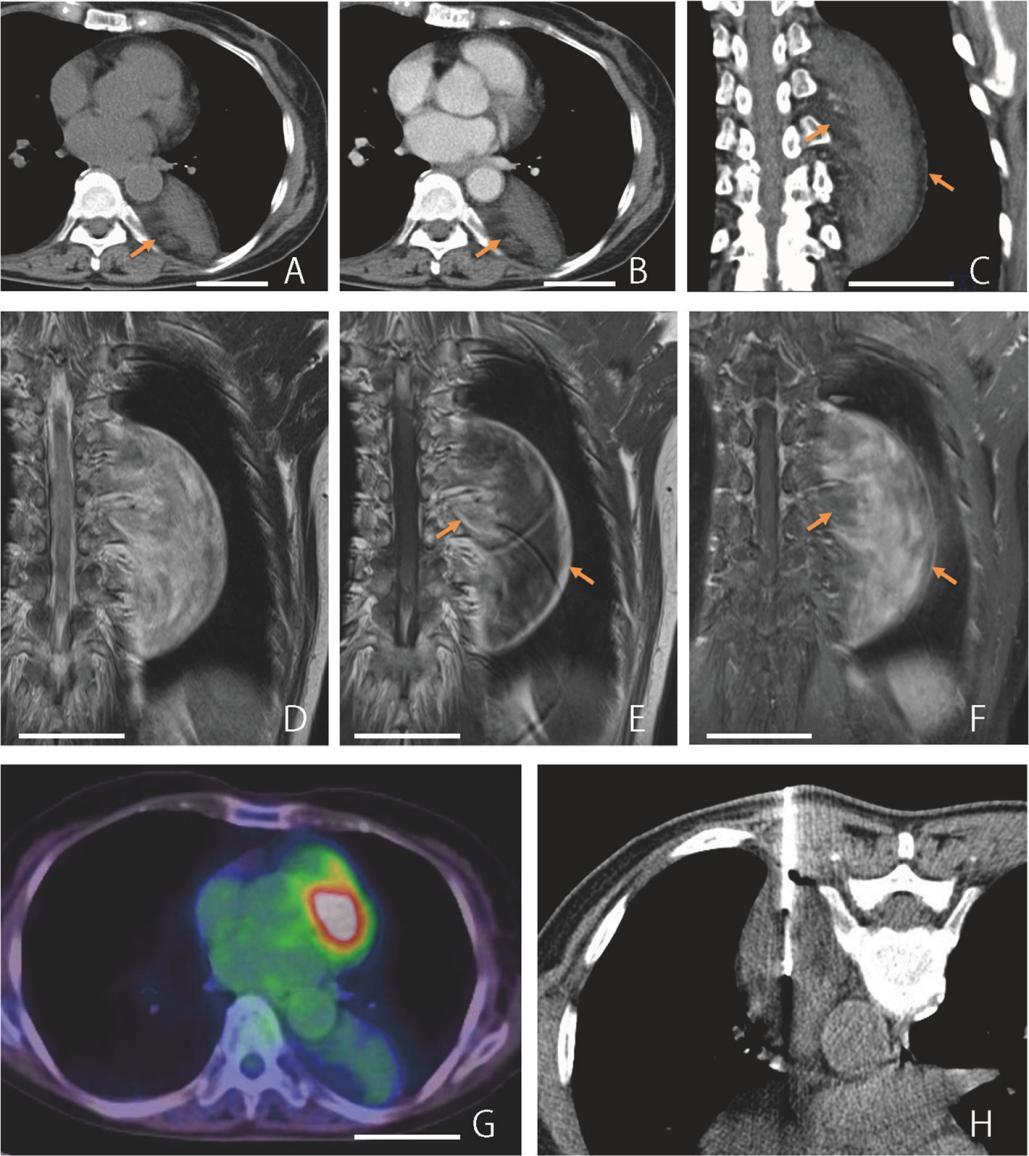
**Radiographic images of the tumor. (A-C)** Computed tomography images. A well-demarcated mass located in the left posterior mediastinum can be seen. The central portion of the tumor shows soft tissue density (27 Hounsfield unit, HU) on precontrasted computed tomography images **(A)**. The late-phase contrast-enhanced computed tomography images **(B, C)** demonstrate slight or mild heterogeneous enhancement (35 HU). **(D-F)** Frontal parallel plane of magnetic resonance images. The tumor shows intermediate to high signal intensity on T2-weighted image **(D)**, low signal intensity on T1-weighted image **(E)**, and slight or mild heterogeneous enhancement on contrast-enhanced fat-suppressed T1-weighted image **(F)**. The tumor appears to be in close contact with several intervertebral foramina. **(G)** 18F-2-fluoro-2-deoxyglucose-positron emission tomography/computed tomography scan at almost the same level as **A** and **B**. **(H)** Computed tomography-guided needle biopsy. The biopsy needle is inserted in the central portion of this mass. The orange arrows seen in **A**, **B**, **C**, **E**, and **F** indicate intratumoral fat tissue. The white bars seen in **A** to **G** represent 5cm.

**Figure 2:**
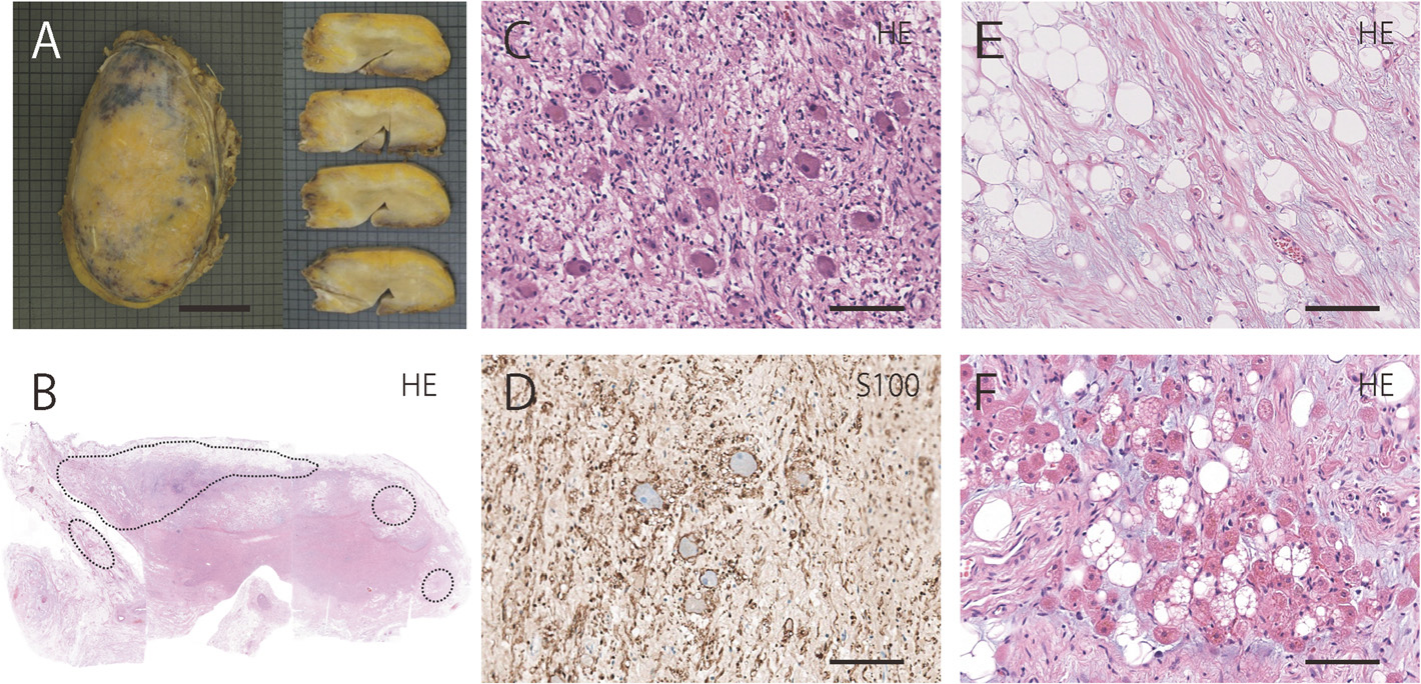
**Macroscopic and microscopic findings. (A)** Macroscopic appearance of the tumor. Yellow fat tissue is mostly present in the periphery. **(B)** Macroscopic figure of maximal, transverse cut surface of the tumor, which probably corresponds to the sections seen in Figure 1A. Brown fat tissue is indicated by circles. **(C)** Histology of the central portion of the tumor. Scattered or clustered ganglion cells and stromal tissue can be seen. Bar, 120μm. **(D)** S100 immunostaining of the central area. Schwann cells and nerve fibers can be observed. Bar, 120 μm. **(E)** Transitional area between the central and peripheral areas of the tumor. A mixture of myxofibrotic tissue and adipose tissue can be recognized. Bar, 120 μm. **(F)** High-power magnification of brown adipocytes. Bar, 120 μm. HE, hematoxylin and eosin staining.

A 66-year-old Japanese woman (height, 145cm; weight, 47kg) was referred for the evaluation of a mass in her left posterior mediastinum. The nodule was noted by her primary care physician about 5 years earlier, and she had also been informed about the presence of an abnormal lesion noted in a chest radiograph around 40 years earlier. She showed no symptoms, and had no significant past medical history. The mass had not changed in size, shape, or location until just prior to her referral. She had undergone computed tomography (CT), which could not exclude the possibility of a well-differentiated liposarcoma due to the presence of intratumoral fat tissue. She was therefore referred for additional investigations. Her physical examination was unremarkable except for mild hypertension. Routine laboratory test results were within normal limits, and levels of tumor markers, including squamous cell carcinoma antigen, carcinoembryonic antigen, cytokeratin 19 fragment, neuron-specific enolase, pro-gastrin-releasing peptide, beta human chorionic gonadotropin, and alpha-fetoprotein were also within normal ranges. Catecholamine levels were not tested. Imaging studies by CT (Figure 1A to 1C) and magnetic resonance imaging (MRI, Figure 1D to 1F) revealed a well-demarcated mass located close to the anterior and left thoracic spine, possibly involving intervertebral foramina (Figure 1C to 1F). The central portion of the tumor showed soft tissue density on precontrast CT images (Figure 1A). In MRI, low signal intensity was seen on T1-weighted images (T1WIs, Figure 1E) with intermediate to high signal intensity on T2WI (Figure 1D). Late-phase contrast-enhanced images by both CT (Figure 1B, 1C) and MRI (Figure 1F) demonstrated slight to mild heterogeneous enhancement. This tumor was rich in fat, especially in peripheral areas, as confirmed by contrast-enhanced fat-suppressed T1WI (Figure 1F). FDG-PET/CT demonstrated high FDG uptake in the central portions of the tumor (Figure 1G). The maximum standardized uptake value (SUVmax) was 2.26, suggesting a tumor with low-grade malignant potential. A CT-guided needle biopsy was performed (Figure 1H), but a definite diagnosis could not be made. Histological analysis of the specimen obtained by CT-guided biopsy demonstrated a mixture of mature adipocytes, spindle cells, and substantial fibrotic component (data not shown). Scanty ganglion cells were present. Immunohistochemical study revealed the existence of S100-positive spindle cells. Peripheral nerve sheath tumor, spindle cell lipoma, and well-differentiated liposarcoma were considered in the differential diagnosis. Since a malignant adipocytic tumor could not be ruled out, surgical resection was performed. During surgery, a well-demarcated mass was seen extending vertically in the posterior mediastinum. This mass was tightly attached to the left side of the thoracic spine from Th7 to Th9. Following intervention, the patient continued to have mild hypertension and was hospitalized for 2 months to control postoperative pain. Three years after the surgery, there is no evidence of recurrence and she remains disease-free.The surgically resected tumor was 12 × 6 × 4cm in size with a yellow and focal black brown appearance (Figure 2A, left). The lesion was well demarcated, although the portion that was resected from the spine showed an irregular surface. The central part of the cut surface was gray in color, and surrounded by a white portion. A yellow fatty tissue was covering the tumoral mass (Figure 2A, right). On microscopic examination, in the gray central portion, ganglion cells were scattered in a fibrous tissue consisting of Schwann cells, nerve fibers, fibroblasts, and collagen bundles (Figure 2C, 2D). Diagnostic neuroblasts could not be identified. Thus, the central portion of the lesion demonstrated features that were typical of ganglioneuroma. The distribution of ganglion cells was consistent with areas showing enhanced FDG uptake by FDG-PET. The highest MIB-1 labeling index was <1% in tumor cells. A myxofibrous stroma with a white appearance was present in the peripheral areas of the ganglioneuroma (Figure 2E). Adipocytes were found towards the periphery, and neither atypical adipocytes nor lipoblasts were seen. Of note, a substantial amount of brown adipose tissue was intermingled in the myxofibrous stroma and white adipose tissue (Figure 2F). This is indicated by the circles in Figure 2B, and accounted for around 20% of the tumor mass. Ganglion cells were not found in adipose tissue. At the area that was tightly attached to the spine, the resected edge appeared to have a positive margin with a fascicular arrangement of Schwannian cells intermingled with white adipocytes. This suggested that the tumor may have involved the intervertebral foramina. The final diagnosis was ganglioneuroma with peripheral replacement by white and brown adipocytes.

On careful retrospective examination of the biopsy specimen with serial sections, only one ganglion-like cell without satellite cells was seen in the fibrotic background. Considering this, the biopsy specimen appeared to be reflective of the peripheral portion of the tumor, even if the needle had been placed in the central portion.

## Discussion

Ganglioneuromas with abundant fat replacement are unusual and only five cases have been reported to date in the English and Japanese literatures [3-7]. Thus, considering the present case, the clinicopathological features of ganglioneuromas with fat replacement show a predilection for middle-aged women (two men, four women, mean age 54 years, range 27 to 73), localization at the posterior mediastinum (five of six cases), and peripheral fat replacement (four of six cases).

Two hypotheses have been put forward regarding the pathogenesis of intratumoral fat tissue. The first involves fatty replacement of atrophied tumor lesions, in which the tumor tissue is gradually replaced by mature fat tissue over time [4]. The second hypothesis involves metaplasia or degeneration of tumor cells into adipocytes [6]. However, at present, the precise pathogenesis of ganglioneuromas with abundant fat replacement remains unclear. In the present case, substantial amounts of brown fat tissue were intermingled in the fatty portion. Brown adipose tissue is normally present in the anterior neck and the thorax, including paraspinal sites [8], rather than in metaplasia from tumor cells. Tumors associated with brown adipocytes have been documented only rarely in the English literature, and when described the tumor has been limited to regions where brown tissue is normally present [9].

In this case, ganglioneuroma was not suspected until after postoperative pathological diagnosis of the surgical specimen. This is because ganglioneuroma is unusual for a fat-containing tumor, and the SUVmax by FDG-PET suggested a low-grade malignancy. Moreover, many diseases may involve fat-containing mediastinal masses, including benign conditions such as extramedullary hematopoiesis [4] and teratoma as well as lipomatous tumors such as liposarcoma and spindle cell lipoma [10], and hibernoma [11]. Schwannoma [12] and neurofibroma [13] may be considered in the differential diagnosis because they can contain adipocytes.

With respect to imaging features, typical CT findings of posterior mediastinal ganglioneuroma [14] include low and homogenous attenuation on precontrast images, occasional intratumoral deposits of punctate calcified materials, and slight to moderate enhancement. The MRI features [14] are homogenous to heterogeneous low signal intensity on T1WI, heterogeneous high signal intensity on T2WI, and mild to marked enhancement on T1WI. While these imaging features are largely similar to those seen in the present case, punctate calcification was absent and the intratumoral fat tissue resulted in a more heterogeneous pattern compared to a typical ganglioneuroma. Even though some reports have suggested that there are no characteristic CT and MRI findings in ganglioneuroma that help to distinguish it from other pathologies [5,15], a recent study has shown that posterior mediastinal tumors with a broad base along the anterolateral aspect of the spine are more likely to be ganglioneuroma rather than schwannoma or neurofibroma [16]. The presence of intratumoral fat tissue and possible involvement of intervertebral foramen restrict the differential diagnoses to ganglioneuroma, schwannoma, neurofibroma, or a lipomatous tumor [5,12,13,17]. Furthermore, the adipocytic elements in schwannoma, neurofibroma, and lipomatous tumors tend to be intermingled randomly or diffusely, which differs from ganglioneuroma with peripheral fat replacement. Thus, the tumor in the present case could have been suspected to be a fat-containing ganglioneuroma considering its radiological features alone. Nonetheless, two reported cases of fat-containing ganglioneuromas [6,7] revealed that intratumoral fat is diffuse or randomly intermingled, and thus histological evaluation is required to obtain a conclusive diagnosis of fat-containing ganglioneuroma. Open biopsy may also be needed because needle biopsy may not sample the central portion of the typical ganglioneuroma component, as in the present case.

Regarding the value of SUVmax seen here (that is, 2.26), it was difficult to rule out well-differentiated liposarcoma because the SUVmax of these tumors has been reported to be around 2.3±1.7 (mean±standard deviation [18]. However, a SUVmax of 2.26 could be considered moderately high if the cut-off value is taken to be 1.8, which is believed to be the optimal value to distinguish benign neurogenic tumors from malignant neoplasms such as malignant peripheral nerve sheath tumor [19]. To date, little is known regarding PET-CT investigations in ganglioneuroma, and only one asymptomatic case with normal catecholamine levels has been reported [20]. In that case, tumor tissue contained abundant mature ganglion cells and increased FDG uptake was seen (SUVmax 2.02). Therefore, together with the present case, this demonstrates that ganglioneuroma may show a low-grade malignant tumor pattern by FDG-PET imaging. Moreover, the presence of brown adipose tissue is known to be linked to false-positive results in FDG-PET [21], which may have contributed to the high FDG-uptake observed in the present case.

## Conclusions

A rare case of ganglioneuroma with peripheral replacement by white and brown fat tissue has been presented, which yielded a false-positive result in FDG-PET by suggesting that the tumor was of low-malignant potential. To the best of our knowledge, this is the first report of a brown fat-containing ganglioneuroma. Ganglioneuroma should be included in differential diagnosis for posterior mediastinal tumors with abundant fat tissue, and clinicians should be aware that FDG-PET of ganglioneuroma can yield a false-positive result by suggesting low-grade malignant potential.

## Consent

Written informed consent was obtained from the patient for publication of this case report and the use of accompanying images. A copy of the written consent is available for review by the Editor-in-Chief of this journal.

## Abbreviations

CT: Computed tomography; FDG: 18F-2-fluoro-2-deoxyglucose; MRI: Magnetic resonance imaging; PET: Positron emission tomography; SUVmax: Maximum standardized uptake value; WI: Weighted image.

## Competing interests

The authors declare that they have no competing interests.

## Authors’ contributions

KY carried out pathological diagnosis on the surgically resected sample, analyzed data, and had a major contribution in writing the manuscript. AY and TA performed clinical examination and surgical treatment. HN performed radiological diagnosis. KN performed clinical follow-up and obtained informed consent from the patient. TF and HK performed pathological diagnosis of the biopsy specimen. All authors have read and approved the final version of the manuscript.
